# A review on biochemical constituents of pumpkin and their role as pharma foods; a key strategy to improve health in post COVID 19 period

**DOI:** 10.1186/s43014-023-00138-z

**Published:** 2023-03-22

**Authors:** Ashiq Hussain, Tusneem Kausar, Sawera Sehar, Ayesha Sarwar, Muhammad Yousaf Quddoos, Jawed Aslam, Atif Liaqat, Tahira Siddique, Qurat Ul An, Samina Kauser, Abdul Rehman, Rizwan Nisar

**Affiliations:** 1 Institute of Food Science and Nutrition, University of Sargodha, Sargodha, Pakistan; 2 Punjab Food Authority, Lahore, Pakistan; 3 Department of Zoology, The University of Lahore, Lahore, Pakistan; 4 Institute of Chemistry, University of Sargodha, Sargodha, Pakistan; 5 Institute of Food Science and Technology, Khwaja Fareed University of Engineering and Information Technology, Rahim Yar Khan, Pakistan

**Keywords:** Pumpkin, COVID 19, Proteins, Oils, Polysaccharides, Vitamins, Minerals

## Abstract

Progression of today’s world has been given setback due to the adversity of a novel, viral, deadly outbreak COVID 19, which raised the concerns of the scientists, researchers and health related officials about the inherent and adaptive immune system of the living body and its relation with healthy diet balanced with pharma foods. Now world is coming out of the destructive pandemic era, the choice of right food can help to build and boost adaptive immunity and pumpkin due to excellent profile of functional and nutraceutical constituents could be the part of both infected and non-infected person’s daily diet. Vitamins like A, C and E, minerals like zinc, iron and selenium, essential oils, peptides, carotenoids and polysaccharides present in pumpkin could accommodate the prevailing deficiencies in the body to fought against the viral pathogens. In current post COVID 19 scenario adequate supply of healthy diet, balanced with pharma foods could play a basic role in boosting immune system of the populations. This review covers the pharmacological activities of pumpkin functional constituents in relation with COVID 19 pandemic. Pumpkins are well equipped with nutraceuticals and functional bioactives like tocopherols, polyphenols, terpenoids and lutein therefore, consumption and processing of this remarkable vegetable could be encouraged as pharma food due to its antihyperlipidemic, antiviral, anti-inflammatory, antihyperglycemic, immunomodulatory, antihypertensive, antimicrobial and antioxidant potential. Need of healthy eating in current post COVID 19 period is very crucial for healthy population, and medicinal foods like pumpkin could play a vital role in developing a healthy community around the globe.

**Graphical Abstract:**

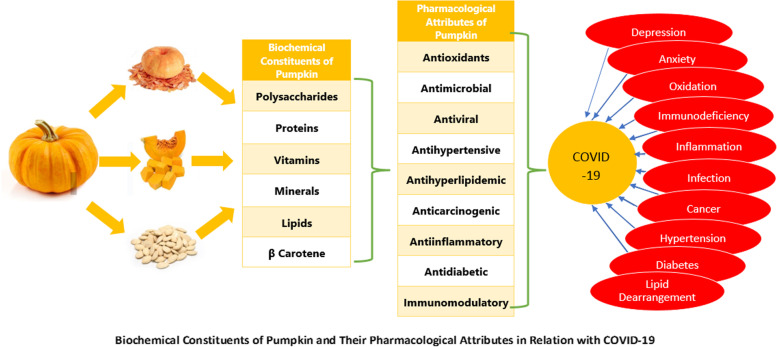

## COVID 19 pandemic, impact on population and need of pharma foods

The COVID 19, a pandemic declared by World Health Organization, is usually caused by a novel corona virus, which normally attacks the people with fragile immune response. Population with weak immunity is more vulnerable towards novel coronavirus. Plant based foods rich in vitamins, minerals and proteins play crucial role in boosting immunity as they promote beneficial bacteria in human body. Food combinations containing immunity boosting nutrients are needed to be explored and marketed in current post COVID 19 period, which can play vital role against common viruses and novel corona virus improving the immunity all around the world (Arshad et al. 2020). This ongoing outbreak is a major global challenge. People with compromised immune system and existing metabolic, respiratory and cardiac problems are more vulnerable to this infection or even death. The pharma foods generated from functional food plants with antiviral and immunomodulatory potential, might not only boost the immune system and cure respiration related infections but also can impose positive health impacts on public (Yang et al. 2020). Beside the development of allopathic drugs and vaccines the use of medicinal foods as dietary supplements or nutraceuticals could be a useful strategy to improve overall health by lowering the risks of other related diseases. Foods derived antioxidants, vitamins, minerals, peptides and metal chelating agents, prevent inflammation and oxidative stress, the conditions which play a major role in advancement of COVID 19. The presence of chronic diseases might enhance the potential risk of defective immune system resulting in attack of novel virus on a fragile defense system of the body (Lammi & Arnoldi 2021).

The consequences of COVID 19 are long term even for those patients who have recovered from this outbreak so it is necessary to visualize in a comprehensive manner the, relationship between COVID 19 and other diseases. It is need of time to develop a relationship between COVID 19, diet, lifestyle and other diseases, this will definitely help the caregivers to attend the patients for better course of medication and recommendations (Toor & Chana 2022). Significant evidences are present that COVID 19 resulted widespread fear, depression, anxiety and mental illness. This pandemic affected billions of people around the globe leading to millions of deaths apart of mental health illness. The impact of this viral disease on global economy shows its destruction on communities, infrastructure and institutions. Now along with the application of vaccine and curing measures it is important to investigate pharma foods, which can boost immune system and mental health of population (Chakraborty 2020). The outbreak of COVID 19 has evolved world health crises and with current rising prevalence of obesity, diabetes and other such complications may lead to persistent derangements in immune system of humans. The role of healthy diet can eliminate these risks by minimizing the immune defections (Zhou et al. 2021).

Nutrition, a key factor affecting humans health must be the key strategy to encounter the attack of COVID 19. That is why the populations with poor diet and bad eating habits have greater chances to be affected by viral diseases especially the novel coronavirus. Food obtained from plant sources are excellent sources of essential vitamins and minerals, the agents responsible for boosting immunity off all the population (Ahmad et al. 2022). Both infected and uninfected persons in the current outbreak of COVID 19 and post pandemic situation, have been advised healthy eating habits in all over the world and inclusion of pumpkin in their diet could be the positive gesture for human health (Abushal 2021). Nutritional foods like pumpkin, rich in most demanding minerals like zinc and iron, could pose beneficial health impacts on populations affected by COVID 19, due to the mediating role of these minerals in oxidation reduction processes (Hussain et al. 2022d).

COVIID 19 survivors have been reported to be involved in different patterns with many physiological impairments, which includes cardiovascular, pulmonary, renal, gastrointestinal, endocrinal, muscular, skeletal, neuro-cognitive, reproductive and hepato-biliary, triggered by FeRD, amplified by HRM, altered mitochondrial function and ACE2/RAAS axis. Natural plant-based antioxidants, immunomodulators, anti-inflammatories and metabolic optimizers play supportive role in nutritional interventions in post COVID 19 complications (Naidu et al. 2022).

## Pumpkin; a pharma food

**Fig. 1:**
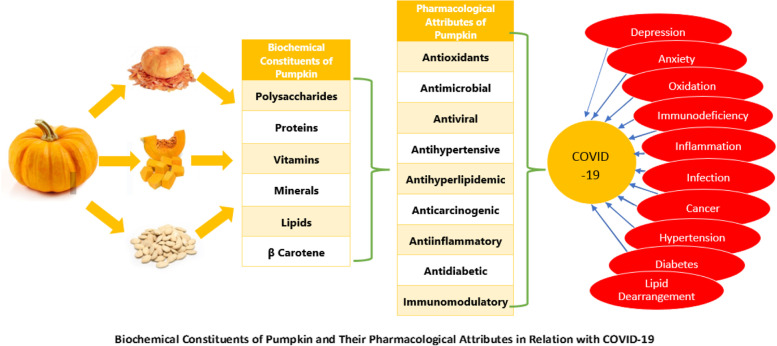
Biochemical constituents of pumpkin and their pharmacological attributes in relation with COVID 19

Fruits and vegetables have important role in human health in aspect of diet as these are healthy source of nutrients such as carbohydrates, proteins, essential oils, vitamins, minerals and fiber. Due to these elements fruits and vegetables possess elite status among food crops. Presence of oxygen radical scavengers like ascorbic acid, calcium, fiber and β-carotene makes fruits and vegetables important part of human diet as these nutrients reduce risk of heart diseases, respiratory diseases, cancer and early aging process. Among fruits and vegetables pumpkin is considered an important vegetable crop due to its nutritional and medicinal uses (El-Aziz et al*.* 2011). A variety of tropical and temperate fruits and vegetables are not explored by the world because of the unawareness of their potential benefits in the market use. Such species of fruits and vegetables due to their medicinal and therapeutic properties have many uses in human consumption (Malik 2010). In context of outbreak of COVID 19 the use of food as medicine has gained importance worldwide and pumpkin containing immunity boosters like omega-3 fatty acids, zinc and selenium is a remarkable food to be used (Parikh & Kumar 2021). Relation of COVID 19 pandemic is very closely related to body immunity and in current situation protection of body from viral diseases can only be done with the use of immunity booster diets. Pumpkin has been included prominently in the selection of vegetables to be preferred during and after COVID 19 pandemic, due to its role as immunity booster (Komarayanti et al*.* 2020). Figure 1 is given for graphical presentation of pumpkin and its relation with COVID 19 treatment.

Pumpkin belongs to the genus *Cucurbita* and family *Cucurbitaceae* which includes cucumber and squash, grown in tropical and subtropical countries. In the world there are three most common varieties of pumpkin named as *Cucurbita maxima*, *Cucurbita moschata* and *Cucurbita pepo* (Lee et al. 2003). Pumpkin is available in many shapes, sizes and colors depending on the variety and climate. Because of the presence of nutritional and health protective polysaccharides in the flesh, and proteins and oils in the seeds, interest in pumpkin and pumpkin-based products, by food processing, agriculture, pharmaceutical and feed industry has been increased. (Sojak and Glowacki 2010). Members of *Cucurbitaceae* family play multifunctional role in both humans and animals’ life. Pumpkins has been traditionally used to cure many diseases and to prepare medicines. Due to presence of nutritional constituents and phytochemicals pumpkins are used as nutritional and medicinal food (Ahmad & Khan 2019). Pumpkin seeds have exhibited great immunomodulatory effects thus these can be used as immunonutrient to boost the immune system against infectious diseases (Iwo et al. 2014). Immunity booster bars have been developed for the athletes, comprised of pumpkin, flax and sunflower seeds, which proved highly effective to fight against the novel virus in this scenario of COVID 19 pandemic (Mishra & Singh 2021).

Presence of fatty acids, polyphenols and prebiotics in vegetables from the *Cucurbitaceae* family makes them striking choice for being used as a nutritional complement during COVID 19. These are known to attenuate the excessive immune response, which may prove to be beneficial in preventing and vindicating COVID 19. These bioactives have been found involved in controlling systematic inflammation and endothelial damage, the two main pathological conditions of COVID 19 prevalence. (Alzaharani et al*.* 2022). Humans’ health can be improved by adding pumpkin in our daily diet as it is an edible food and can provide various health benefits. Pumpkin plays important role in human health by acting as medicinal food because it has potential of anti-diabetic, anti-oxidant, anti-microbial, anti-carcinogenic and anti-inflammatory agent. Some other health benefits of pumpkin have also been reported by different scientists which include hypotensive, inhibition of kidney stones and blood coagulatory effects. For the treatment of diseased conditions, pumpkin role as synergistic and no change effects is very important (Yadav et al. 2010). Pumpkin seed oils have been recognized as good source of phenolics (ferulic acid, syringic acid, chlorogenic acid, *p*-coumaric acid, tyrosol, vanillic acid, vanillin, luteolin and sinapic acid), whereas total phenolic contents in pumpkin seed oils ranged from 24.71 to 50.93 mg/gallic acid equivalent/kg oil. Pumpkin seed oils oxidative stability was calculated about 4 h and antioxidant capacity was found 62%, measured by DPPH radical scavenging method (Andjelkovic et al. 2010). Significant amount of minerals (potassium, iron, zinc, copper, magnesium, selenium and phosphorus) and phytochemicals (α tocopherol, β tocopherol, γ tocopherol, β sitosterol, stigmasterol, squalene and β carotene) have been quantified from different varieties of pumpkins, and extracts from pumpkin were found involved in antimicrobial activities against different bacterial and fungal strains (Singh & Kumar 2022). As functional compounds in pumpkins and the secondary metabolites in plants, the positive effects of polyphenols have been remained obvious, including high antioxidant capacities and the mitigation potential of chronic diseases and certain cancers (Yang et al. 2022). Pumpkins contain sufficient amounts of dietary fiber due to which their glycemic index are low, that’s why pumpkin has been used traditionally to cure diabetes. Pumpkin powder incorporated chocolates were found effective in positive mood changes during studies on humans (Shahidan et al.^2017^). Pumpkins are considered excellent source of provitamin A carotenoids, which are very helpful in prevention of vitamin A deficiency (Kim et al. 2012). Pumpkins are excellent source of phytochemicals and also possess significant amounts of protein, fat, fiber and tocopherol, which have been found abundantly in pumpkin seeds. All these nutrients have nutritional qualities in the foods as studies in the past has elaborated the use of fiber as antidiabetic material (Brennan & Tudorica 2008).

**Fig. 2:**
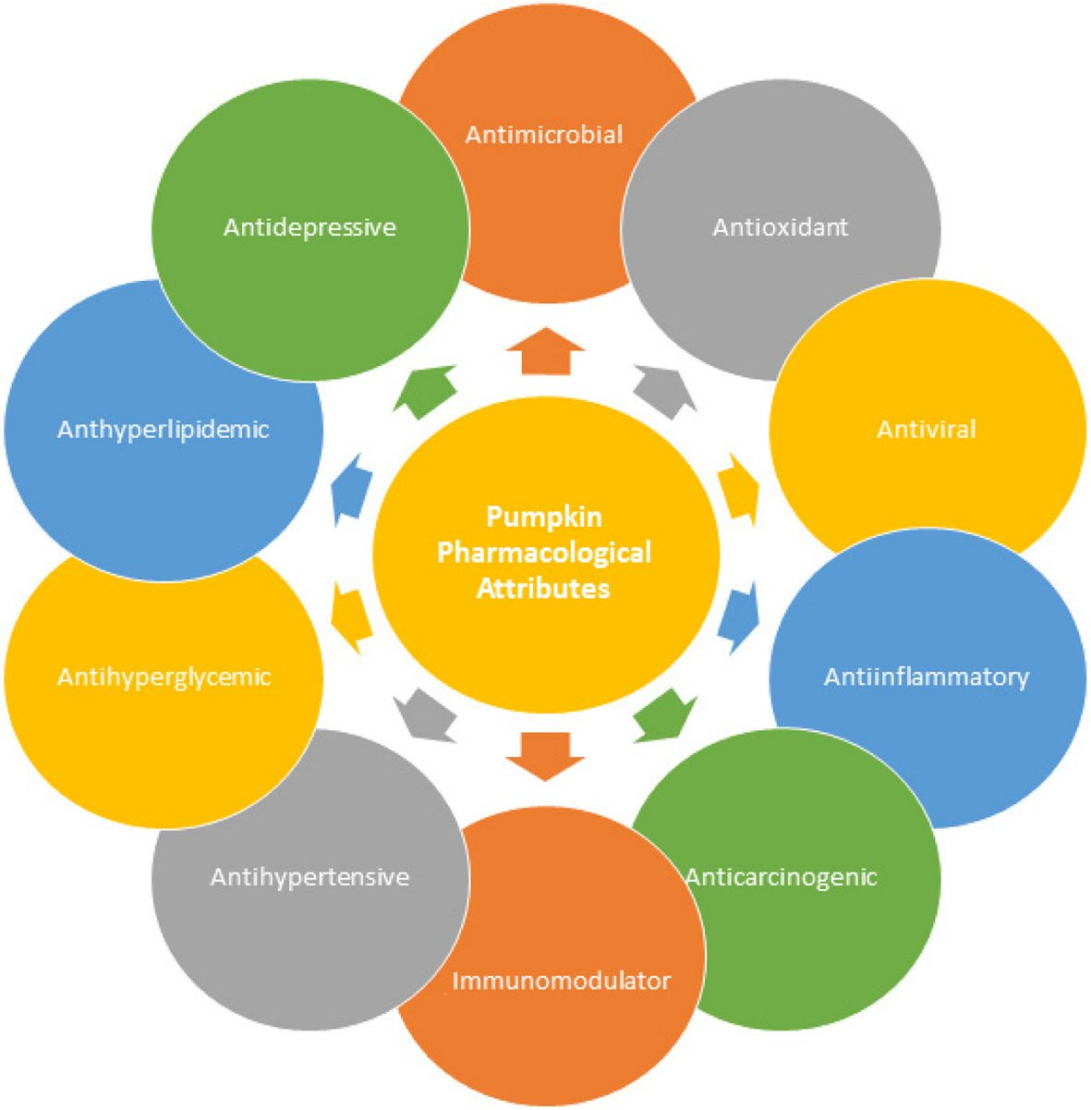
Pharmacological attributes of pumpkin

Drying is a method, which is among the most conventionally adopted at every scale process for the preservation of perishable foods. A variety of different range of dried products are either consumed directly or utilized for the production of different products by food industry. Drying may be effective to preserve the nutritional status of fruits and vegetables (Benseddik et al. 2019). Pumpkin dried powder has been utilized in instant drink packed with natural compounds acting as a neutralizer of the free radicals in the body, which act as triggers for diseases. Carotenoids, especially β carotene is a natural antioxidant present in pumpkin, and has proved helpful in curing cancers and neurodegenerative diseases (Surya et al. 2021). During Halloween, huge quantities of pumpkin waste are produced and people are well aware about the nutritional importance of this waste, which can be converted into different recipes in the era of COVID 19 as populations may face food scarcity. If properly implemented this technique will prove useful in providing people healthy food (Surucu-Balci & Berberoglu 2022). Pharmacological attributes of pumpkin have been summarized in Fig. 2.

Pumpkin’s popularity as medicinal plant has sought the attention of various investigators. Significant proves from different experiments regarding bioactivities led to analysis in various animal models, studies about structure of cell and clinical trials aimed to analyze pharmacological properties of pumpkin. Pumpkin has number of phyto-constituents that have been classified in to different groups. Moreover, basic medicinal characteristics of pumpkin constituents are their role as anti-inflammatory, antioxidant and anti-carcinogenic agents in animals (Adams et al. 2011). Diabetes is very closely related risk factor for COVID 19 patients. Fruits and vegetables are comprised of natural constituents like carotenoids that can block digestion of carbohydrates and enzymes minimizing the risk of diabetes (Surya & Marpaung 2022). Active-hypoglycemic characteristics that can be obtained through pumpkin are seed oils, pectin, hypoglycemic proteins and non-pectin polysaccharides (Caili et al. 2006). Polysaccharides that are protein bounded have potential against diabetes as it has ability to enhance glucose tolerance, raise serum tolerance level of glucose and minimize the level of blood glucose (Quanhong et al. 2005). Vegetables like pumpkin, which are loaded with antioxidant components such as Vitamin C, A, E, zinc and β carotene have high impact on COVID 19 patients. Instead of consumption of fast foods possessing bad cholesterol and saturated fats, these types of vegetables improve innate and adaptive immune system of the body (Tanmoy et al. 2020). Pumpkin is one of these medicinal plants, which is cultivated all over the world and its fruits are traditionally used for human consumption to cure chronic diseases. Experiments on streptozotocin-induced diabetic rats, with application of pumpkin extracts provided effective results to lower down the blood glucose level (Xia & Wang 2007). Study conducted on extracts of pumpkin peels and pulp proved that pumpkin components are capable of exhibiting strong antimicrobial and antioxidant activities (Asif et al. 2017). *Curcubita* (pumpkin) is an herbaceous vine attached, edible and heat sensitive vegetable, well-known due to its food and medicinal value, which has been found associated with macro and micro nutrients present abundantly, and relatively low amounts of antinutrients (Kaur et al. 2019). Along with family of drugs, which includes vaccines and certain inhibitors the role of functional food components emerged from plants is very crucial in combating COVID 19. Natural plant-based bioactives including antioxidants, immunomodulators, anti-inflammatories, antimicrobials, anticarcinogenic and metabolic optimizers have been found involved in promoting body functions by playing supportive role in nutritional interventions resulted due to the COVID 19 (Naidu et al. 2022).

Pumpkin based soups and creams could be the easiest prepared possible foods for COVID 19 patients as pumpkins are rich source biologically active components, which can provide relief from disease symptoms (Perez-Alvarez et al. 2020). Pumpkins oily contents consist of omega 6 fatty acid mainly, which has various biological applications as well as considerable antioxidant activity along with hypoglycemic and anti-inflammatory features. In clinical trials it was proved that oral treatment of chemically induced diabetic rats with omega 6 fatty acids, restored the anti-oxidant status to normal range in various tissues (Suresh & Das 2003). Pumpkin is one of the well-known medicinal plants and considered as functional food promoting human health when consumed in safe manners. The presence of a number of biologically active components such as polysaccharides, proteins, peptides, sterols, fixed oils, and para-aminobenzoic acid make pumpkin as a beneficial food for humans (Caili et al. 2006). Pumpkin seeds are also valued for presence of pumpkin seed oil containing prominent amount of essential fatty acids. Along with seed oil pumpkin seeds are also a good source of proteins containing high values of essential amino acids (Glew et al. 2006). A component named as D-chiro-Inositol has been recovered from pumpkin which is an insulin secretor and sensitizer (Xiong 2000). In clinical trials it has been proven that in patients with depression, when treated with pumpkin and β carotene, increased level of serotonin and norepinephrine was found in brain, the hormones responsible to cure depression (Kim et al. 2016). Proper choice of diet filled with essential micronutrients can help to strong the adaptive immunity of the body by avoiding attacks of pathogens. The role of healthy diet cannot be neglected in fighting against infectious diseases (Sharma, 2020). Pumpkin fractions like peel, flesh and seeds are capable of imposing in vitro pharmacological role in animals and humans as these constituent parts are loaded with bioactives (Hussain et al. 2022a). Functional constituents present in pumpkin and their pharmacological activities related to COVID 19 pandemic have been presented in Table 1.

**Table 1:** Functional constituents present in pumpkin and their pharmacological activities related to COVID 19 pandemic

Sr. No	Functional constituents	Pharmacological activities related to COVID 19
1	Vitamin C	Destroys free radicals in the body and boost immune system
2	Vitamin E	Act as antioxidant by scavenging the free radicals in the body
3	Vitamin D	Encounter the viral infections, especially respiratory tract infections which are common in COVID 19 outbreak
4	β carotene	Powerful antioxidant, reduces inflammation by increasing the production of diseases fighting cells in the body
5	Zinc	Increases the production of white blood cells in body, the agents defending body against viruses
6	Iron	Necessary for normal functioning of biochemical functions in the body, homeostasis risk of infection
7	Selenium	Boost both innate and adaptive immune system
8	Polysaccharides	Reduce level of oxidative stress in cells and tissues and play role as antioxidant, antimicrobial, antiviral, antidiabetic and antitumor
9	Proteins (peptides, enzymes)	Act as antimicrobial, inhibit growth of melanoma, counteract intoxication,
10	Lipids (fats, essential oils, fatty acids)	Play role as antioxidant, anti-inflammatory, immunomodulatory, anticarcinogenic and hypotensive

## Pumpkin constituents possessing pharmacological activities in relation with COVID 19

### Polysaccharides

Plant derived polysaccharides from pumpkin reduces the oxidative stress level in cells and tissues and could decrease the diabetes symptoms in animals as well as humans (Fang et al. 2021). Polysaccharides extracted from pumpkin have been found involved in several pharmacological activities including antiviral, antioxidant, antitumor, immunoregulatory, hepatoprotective and hypoglycemic potential (Ji et al. 2021). A low molecular weight polysaccharide from pumpkin with 3.5 kDa weight has been isolated exhibiting strong antioxidant and hypoglycemic potential, it also exerts good inhibition rates of α glucosidase and α amylase (Li et al. 2021). Pumpkin polysaccharides possess better antioxidant activity so can be used as antioxidant foods (Chen et al. 2020a, 2020b). Polysaccharides in pumpkin peel and pulp exhibit hypoglycemic activity in animals by lowering plasma lipids concentrations (Caili et al. 2006). A study conducted by Zhao et al. (2014) on hypoglycemic effect of pumpkin polysaccharides extracted from pumpkin witnessed that the pumpkin polysaccharides can reduce the risk of diabetes. From pumpkin fruits pulp a variety of polysaccharides have been recovered having hypoglycaemic potential. Because of low energy value dietary fiber obtained from pumpkin pulp is very helpful in lowering blood glucose level (Jun et al. 2006). A neutral polysaccharide isolated from pumpkin exhibited immunomodulatory activities toward macrophages (Huang et al. 2021).

Pectic polysaccharides possess anti-infective and anti-inflammatory activity which is directly related to COVID 19 outbreak as coronavirus attacks mainly respiratory system through infection and inflammation (Wang et al.^2021^). Quanhong et al. (2005) reported that hypoglycaemic effect of pumpkin is due to protein bound polysaccharides which were isolated from water soluble substances of pumpkin fruits. These protein-bound polysaccharides from pumpkin were isolated and evaluated on alloxan induced diabetic rats to check the hypoglycaemic potential, it was observed that level of blood insulin was increased, level of blood glucose was reduced and these alloxan induced diabetic rats showed improved tolerance of glucose. Pumpkin polysaccharides are non-specific immune boosters that employ various monitoring functions on the immune system resultantly improving the body immune system to fought against the viral and infectious diseases (Chen et al*.* 2018).

Pumpkin flesh polysaccharides are involved in antioxidant activities (Chen & Huang 2019). Starches isolated from pumpkin flesh were used to develop edible films (Pajak et al*.* 2019). Research conducted by Chen et al. (2020a, 2020b) provide useful information for understanding the inhibitory effects of polysaccharides from pumpkin flesh, on LDL oxidation.

### Vitamins and minerals

Role of vitamins and minerals in enhancing immune response has brought the attention of the scientific community in this post COVID 19 era and pumpkin as a rich source of vitamin A, C and E can play vital role in helping immune system to encounter viral infections (Dhok et al. 2020). Vitamin E (tochopherol) which is an antioxidant is found in high contents in pumpkin seeds and pumpkin seeds oil which contains substantial amount of vitamin E is a good part of Japanese diets (Tokudome et al. 1999). Chang et al. (2004) demonstrated that serous and hepatic activities of glutathione peroxidase and superoxide dismutase are significantly increased by administration of pumpkin fruit extracts in mice and also concentration of malonaldehyde is reduced to a significant level. The activity of glutathione peroxidase and superoxide dismutase could increase by pumpkin polysaccharides in the serum of tumor containing mice. Malonaldehyde content are also reduced (Guo-hua 2000). Pro-oxidative pathways are activated as a result of viral infections, vitamins which act as antioxidants reduce the risk of inflammation ultimately protecting the organs from failure. Vitamin intake lowers the oxidative stress, alleviates cytokine storm ultimately decreasing disease severity specially in viral infections (Pisoschi et al. 2022).

Pumpkins utilization has different forms, like fresh pumpkin, boiled, cooked, in the form of powders, extracts, purified bioactives and pumpkin based functional and pharma food products. Excellent nutritional profiles of pumpkin parts are the strong witness of utilization of pumpkin by every age of people in every region of the country as this will lead to decrease in burden on health sector. Specially in current corona virus situations healthy need of eating balanced with pharma foods like pumpkin could play an important role in boosting immunities of the populations and communities so that attack of deadly and viral diseases could be prevented (Hussain et al. 2022c).

Pumpkins are excellent source of an important mineral Zn, which plays a vital mediating role in activation of enzymes and in this current situation of pandemic consumption of pumpkin can promote antioxidation in the living body thus restricting the attack of viral diseases (Hussain et al., 2021). Powders and pastes made from pumpkin rich in micro minerals especially Zn can be used as a food to mitigate food and nutritional challenges in current post COVID 19 pandemic situations (Hosen et al., 2021). Steiner-Asiedu et al. (2014) made research on nutrient composition and protein quality of four different species of pumpkins and his results for proximate composition were: moisture, crude protein, fat and ash 5.44–6.66, 30–36, 44–58 and 3.18–4.90% respectively. The amount of crude fiber was found less than 2.5%. On mineral analysis the higher values of Zn, Cu and Fe were found which are 5.1–7.1, 1.4–7.9 and5.6–8.5 mg/100 g respectively. Mineral’s analysis of pumpkin seeds cultivated in Zimbabwe declared that these have high nutritional contents like protein, fat, fiber, carbohydrates and minerals like Zn, Fe, Ca, Mg and P. These seeds are good alternate of food with less nutritional values (Kwiri et al. 2014). Zinc is a very important mineral responsible for enhancing metabolism function and immune system. Pumpkin seeds derived peptides bonded with natural zinc present in pumpkin seeds are excellent nutrients responsible for well-maintained immune system (Lu et al. 2021). Chocolate fortified with zinc from pumpkin seeds has been developed to overcome zinc deficiencies in COVID 19 times as a dietary supplement (Suchitra et al. 2021).

### Proteins, peptides and enzymes

Peptides from pumpkin seeds after digestion in digestive tract act as anti-COVID 19 agents, therefore these can be consumed on daily basis for the prevention and management of this outbreak. Molecular docking study has revealed that from seed proteins during digestion,1593 peptides were released out of which, 36 were of high gastrointestinal absorption. These bioactive peptides could bind the catalytic sites of the viral proteins. Pumpkin seed protein derived peptides possessed more strong affinities toward binding of COVID 19 spike proteins (Wong et al. 2021). More proteins were isolated from pumpkin seeds which were involved in inhibition of melanoma proliferation (Xie 2004). Xia et al. (2003) isolated a novel ribosome-inactivating protein (RIP) called moschatin from mature pumpkin seeds and successfully prepared a novel immunotoxin moschatin-Ng 76 which more efficiently than free moschatin inhibits the growth of specific melanoma cells. Hou et al. (2008) isolated a novel type 1 ribosome-inactivating protein designated cucurmosin from the sarcocarp of pumpkin (C. *moschata*). This protein exhibits a strong cytotoxicity to three cancer cell lines of both murine and human origin beside rRNA and N-glycosidase activity.

A peptide with molecular weight 8 kDa from pumpkin seeds was isolated and observed to inhibit *Botrytis cinereal, Fusarium oxysporum* and *Mycosphaerella arichidicola* at a dose of 375 µg (Vassiliou et al. 1998). From fresh brown pumpkin seeds two proteins named as α-moschin and β-moschin with a molecular weight of 12 kDa were isolated, which showed translation-inhibition activity with 50% inhibitory concentration of 17 µM and 300 nm respectively (Xiong 2000). A purified protein with molecular weight of 28 kDa from pumpkin fruit exerted a significant antifungal effect against the growth of *Fusarium oxysporum* at a concentration of greater than 2 Mm in an agar disc plate media. The same protein exhibited a synergistic effect with nikkomycin, a chitin synthase inhibitor, as a growth inhibitor of *Candida albicans* (Ng et al. 2002). Pumpkin seeds also contain antimicrobial protein among these are three basic proteins MAP2, MAP4 and MAP11 with molecular weight 2.2 kDa, 4.6 kDa and 11.7 kDa respectively are observed having inhibitory effect on the growth of yeast cells. MAP11 was found to be the most effective inhibitor of yeast cells. The growth of Gram-negative bacterium E. *coli* was not inhibited by MAP2 and MAP4 (Cheong et al. 1997). It has been reported that pathogenic fungal proteases are inhibited by phloem exudates from pumpkin fruits which indicates antifungal spectrum of pumpkin (MacGibbon & Mann 1986). Recently a new protein named as Pr-1 was isolated from pumpkin by Park et al. (2010) which exhibits strong antifungal effect without any harmful or toxic effect on human erythrocytes. This protein is quite stable towards high temperature up to 70 ^0^C but no inhibitory effect towards growth of E. *coli* and *Staphylococcus aureus*. From all these data it can be concluded that pumpkin can be consumed by humans as an antimicrobial agent as it will protect human beings from infectious diseases caused by pathogenic micro-organisms.

In case of intoxication resulted from carbon tetra chloride the administration of pumpkin seed proteins activity levels of alanine transaminase, aspartate transaminase, lactate dehydrogenase and alkaline phosphatase were significantly reduced, so it was concluded that administration of these proteins was effective in protein malnutrition condition and its adverse effects (Nkosi et al. 2005). Pumpkin seeds proteins are also involved in blood clotting process as it could have inhibitory effects on trypsin and activated Hageman factor (Krishnamoorthi et al. 1990). Pumpkin flesh, peel and seeds powders have sufficient crude fiber and proteins, especially pumpkin seeds proteins with essential amino acids and peptides, which are responsible for promotion of healthy body functions in humans (Hussain et al. 2022b).

Steiner-Asiedu et al. (2014) made research on nutrient composition and protein quality of four different species of pumpkins. The PER and NPU values were in the range of 0.75–1.36 and 46.10–69.10 respectively which clears that all these varieties had good protein quality. Amino acid studies of some pumpkin verities revealed that amino acid profile contain high amount of glutamic acid ranging from 33.03 to 34.76 g/100 g protein (Al-Anoos et al. 2015). An amino acid (3-amino-3-carboxypyrrolidine) called as Cucurbitin present in Cucurbitaceae family and their seeds is used to kill intestinal parasites such as tapeworms and roundworms (Gill et al. 2011). Various parts of pumpkin plants contain various antibiotic components including antifungal agents. Some of these antifungal agents are proteins such as α and β-moschins, myeloid antimicrobial peptide and a peptide (MW 8 kDa) have been isolated and characterized from pumpkin plants (Vassiliou et al. 1998).

### Lipids, fatty acids and essential oils from pumpkin

Pumpkin seed oil extracts in the form of microemulsion, when used for the treatment of COVID 19 patients expressed promising results due to their anti-inflammatory, antioxidant and immunomodulatory effects. Nanostructure of squalene has been tested in clinical trials for its efficacy on COVID 19 patients and at the end of trials blood tests revealed that no adverse effects of squalene were induced on blood cells, suggesting the potential use of squalene for treatment of COVID 19 (Ebrahimi et al*.* 2022). Pumpkin seed oil contains different carotenoid pigments which provide different health benefits, major among these is anti-carcinogenic effect (Jian et al. 2005). The risk of breast, gastric lung and colorectal cancer is reduced to a greater extent by utilizing diets high in pumpkin seeds (Huang et al. 2004). Prevention of prostate cancer is linked with carotenoid pigments present in pumpkin fruits (Binns et al. 2004). Fahim et al. (1995) conducted his research work on anti-inflammatory activity of pumpkin and reported that just like indomethacin (a well-known anti-inflammatory medicine) pumpkin seed oil inhibits adjuvant-induced arthritis in rats. They concluded that for the treatment of inflammatory diseases supplementation of natural substances with standard drugs can provide drug interaction effects; no change effects which are more synergistic and antagonistic. Natural substance from pumpkin when used with standard dug as a formulation will boost the anti-inflammatory action. Al Zuhair et al. (2000) suggested that pumpkin seed oil possess hypotensive activity. They tested pumpkin seed oil with standard hypotensive drug felodipine, a Ca antagonist and suggested that pumpkin seed oil has a very good drug interaction effect. They used hypertensive animal models for this research and also tested pumpkin seed oil as supplement with an angiotensin-converting enzyme inhibitor named as captopril to check the hypotensive potential. Pumpkin seeds oil which is well known for its nutty taste and smell, greenish color, contains α linolenic acid and oxylipin products. Bioactive phytoprostanes and bioactive phytofurans have also been found in pumpkin seed oil (Vigor et al. 2022).

Pumpkin seeds oil are good source of edible oil and protein as they contain 41.59% fat and 25.4% protein contents. Further proximate analysis of pumpkin seeds reported moisture 5.2%, crude fiber 5.34%, total ash 2.49% and carbohydrates 25.19% (Ardabili et al. 2011). According to Achu et al. (2005) pumpkin fruit seeds from different regions of Cameron can be utilized as oil and protein sources as they contain 28–40% protein contents, 44–53% fat contents and 7–10% carbohydrate contents. Al-Anoos et al. (2015) studied the chemical composition of some Chinse and Egyptian pumpkins. The results were: crude fiber 4.12 to 4.69%, total lipids 35.2 to 41.95%, crude protein 34.19 to 39.75% and total carbohydrates 4.8 to 10.96%. Achu et al. (2013) reported that all Cucurbitaceae family oilseeds are rich source of some essential amino acids and gives protein digestibility.

Karanja et al. (2013) proved that pumpkin seeds are rich source of crude oil, crude protein, crude fiber and carbohydrates. They studied nutritional composition of Cucurbitaceae family seeds harvested from selected regions of Kenya and found that seeds from all varieties of pumpkins are rich in oil, protein and fiber. The fatty acid profile of pumpkin seeds is rich in polyunsaturated fatty acids, which is very similar to sesame, soya bean and sunflower oils. Proximate analysis of different pumpkin seeds varieties resulted crude protein (14.05–33.29%), crude fiber (11.69–24.85%), crude fat (31.9–41.37%) and carbohydrates (8.66–27.35%). Fatty acids profile from the extracts of pumpkin seeds cleared those unsaturated fatty acids were in high ratio and among them the most abundant were linoleic acid (26.18–81.21%), oleic acid (15.56–30.79%), palmitic acid (1.16–20.81%) and stearic acid (0.16–5.56%). Recent research by Montesano et al. (2018) revealed that pumpkin seed oils due to presence of monounsaturated fatty acids, polyunsaturated fatty acids, phytosterols and carotenoids can be used as functional and preservatives ingredients in nutraceutical and cosmetic industry. These seed oils can also be used into different food formulations for the benefit of mankind. Pumpkin seed oils are good source bioactive components possessing excellent antioxidant activities (Irmawati et al. 2022).

Further clinical work is required to be undertaken before those isolated, purified compounds can be marketed. However, in the developing countries, to cut down costs, intake of pumpkin fruit in form of vegetable should be encouraged that also shown clinically effective. To maintain healthy body with properly working immune system consumption of pumpkin on daily basis must be encouraged in this current period of post COVID 19.

### β carotene

Carotenoids especially β carotene is thought to prevent diseases mainly due to its antioxidant role as it can be converted in to Vitamin A in human body. These carotenoids cannot be synthesized by our body and only source is diet or supplements. In current COVID 19 outbreak diet rich in carotenoids should be appraised for people of all age of groups in the world (Khalil et al. 2021). Pumpkins are grown at vast areas of tropical and subtropical states where these are consumed as vegetable in boiled or steamed form or these are processed to make soup and curry. Yellow or orange color of pumpkins are due to high level of β-carotene contents present in them (Kandlakunta et al. 2008). Pumpkin powder is a concentrated source of β-carotene and it is a store house of phytochemicals which play very important role for improvement of human health. Pumpkin powder is used in different bakery products for its flavor, typical deep yellow to orange color, sweetness and dietary fiber (Das & Banerjee 2015). Plants with yellow to orange color contain high amounts of β-carotene which is a good source of vitamin A. Consumption of β-carotene containing foods on regular basis prevents cancer, skin disorders and eye disorders in humans (Bendich 1989). Vitamin A deficiency disorders can be prevented and nutrition level of individuals can be improved by incorporation of β-carotene rich foods into daily diets (Siems et al. 2005). Carotenoids play important role in prevention of cancer which is their special physiological functionality along with provitamin-A activity. Contents of carotenoids in the food is an area of research for scientists which is needed to be expanded (McCann et al. 2004). Pumpkin peel contains the largest amounts of total carotenoids (β-carotene, β-carotene, violaxanthin, neoxanthin, all-trans-lutein) and isomers (9-cis β-carotene, 13-cis-β-carotene, β-ionon, α-ionon, dihydropseudoionon, β-cyclocitral, tocopherol and tocotrienol) among all fractions, but in different concentrations in different varieties. Range of these carotenoids and isomers in pumpkin was 0.97–112 µg/100 g (Jakob & Elmadfa 1996). β carotene is among the molecules, which are healthy candidates for the development of new drugs to combat COVID 19 (Costa et al. 2021).

Pumpkin is an important source of healthy nutrients which includes minerals, dietary fiber, ascorbic acid, carotenoids, tocopherols and phenolics. β-carotene present in pumpkin is an anti-inflammatory agent as it decreases the skin cancer which occurs due to sunlight whereas α-carotene is considered responsible to prevent aging, growth of tumor and preventing the chance of developing cataracts in eye. (Jacobo-Valenzuela et al. 2011). Pumpkin fruits when fully ripe are sweet with orange to yellow flesh and are rich in β-carotene; a precursor of vitamin A. A variety of antioxidants such as vitamin E, C, K and B_2_ have been reported in pumpkin fruit along with carotenes and phenolic compounds (Chanwitheesuk et al*.* 2005). Carotenoids can be the potential agents to limit the devastating effects of COVID 19 (Ghasemnejad‐Berenji 2021). Pumpkin and its functional constituents like carotenoids, especially β carotene have been found present in pumpkin based functional food products, which can be served to the both normal and affected populations for normal smooth functions (Hussain et al. 2022).

**Fig. 3:**
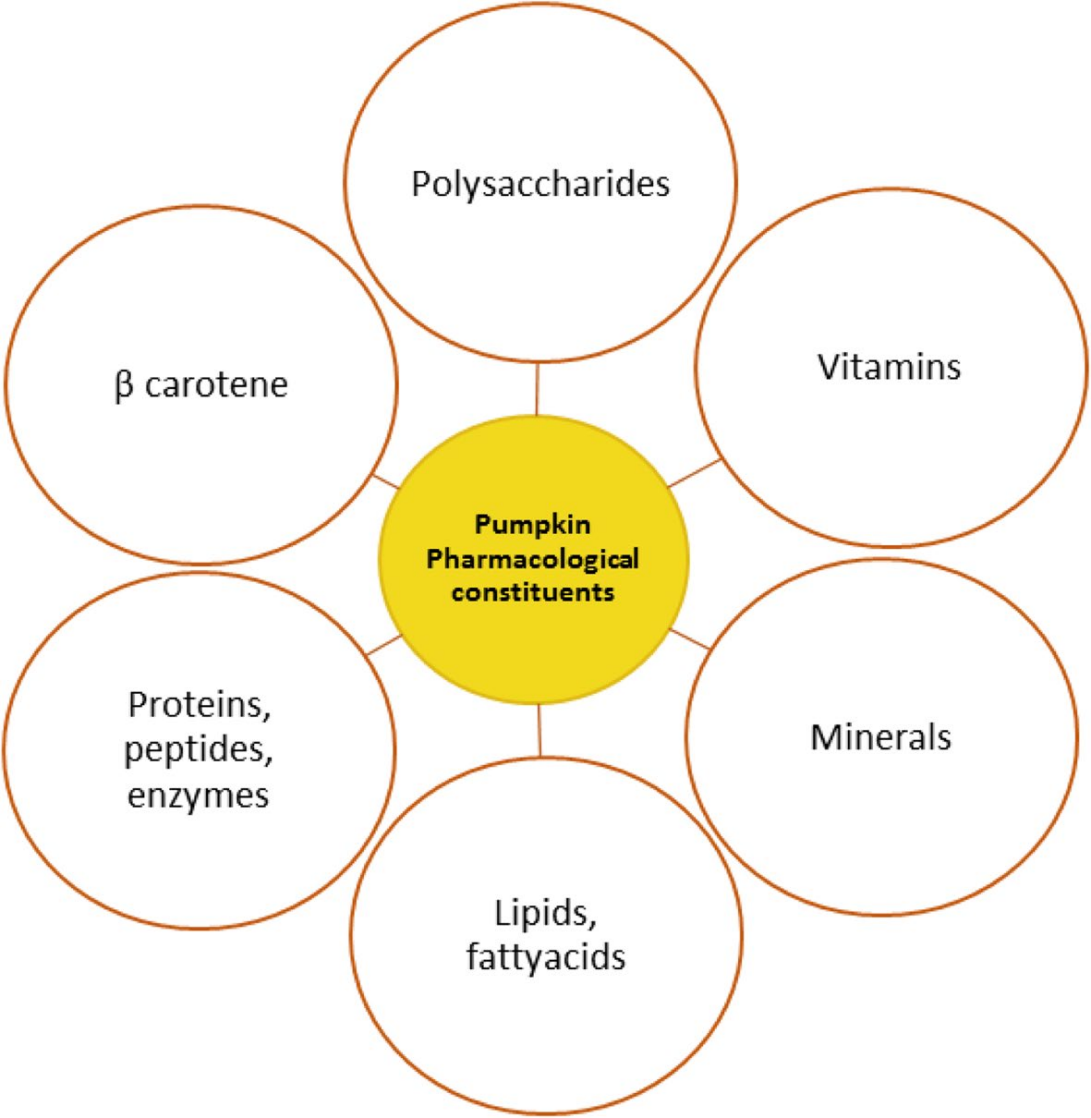
Functional and pharmacological constituents of pumpkin

Pumpkin fruits are an important source of carotenoids and these carotenoids in the form of pro-vitamin A play important role in human nutrition. Skin diseases, eye disorders and cancer can be prevented by using pumpkin-based diets as these diets are rich source of carotenoids. Incorporation of pumpkin as an ingredient and source of β-carotene in a variety of food products is considered very effective towards vitamin A related health disorders and it is also very much cost-effective approach. For the development of nutraceuticals and value-added food products incorporation of pumpkin has gained great interest due to its anti-inflammatory, antioxidant, antidiabetic and anticarcinogenic activities. Pumpkin fruit could be consumed as a source of different bio-actives promoting human health (Dar et al. 2017). In order to tackle the negative health impacts of COVID 19 along with positive attitude, consumption of plant-based foods enriched with immune strengthening nutrients like carotenoids must be encouraged (Muscogiuri et al. 2020). Functional and pharmacological constituents of pumpkin have been presented in Fig. 3.

## Conclusion

Along with allopathic drugs and vaccines, healthy eating compromised of pharma foods should be the key strategy to counter the attack of diseases especially the COVID 19. Populations with poor eating habits have been badly affected by the attack of novel coronavirus due to their weaker immune system. Pumpkin has been proved an excellent pharma food, possessed with remarkable amounts of functional and nutraceutical constituents responsible for multifunctional roles in human body. Proteins, polysaccharides, oils, vitamins, minerals and phenolic compounds present in pumpkin fruits exhibits immunomodulatory, anti-inflammatory, antioxidant, antimicrobial and antiviral activities. Need of the time is to incorporate these constituents in humans’ daily diet to maintain a well-balanced body equipped with boosted immune system.

## Data Availability

Data relevant to this study can be provided upon request.
